# Anti-parasitic Peptides from Arthropods and their Application in Drug Therapy

**DOI:** 10.3389/fmicb.2016.00091

**Published:** 2016-02-05

**Authors:** Ariane F. Lacerda, Patrícia B. Pelegrini, Daiane M. de Oliveira, Érico A. R. Vasconcelos, Maria F. Grossi-de-Sá

**Affiliations:** ^1^Plant-Pest Interaction Laboratory, PBI, Genetic Resources and Biotechnology, Brazilian Agriculture Research CorporationBrasilia, Brazil; ^2^Post-Graduation Program in Biochemistry and Molecular Biology, Federal University of Rio Grande do NorteNatal, Brazil; ^3^Post-Graduation Program in Pharmaceutical Sciences, Faculty of Health Sciences, University of BrasiliaBrasilia, Brazil; ^4^Department of Health Sciences, Integrated College of Educational Union’s Central PlateauGama, Brazil; ^5^Post-Graduation Program in Genomics Science and Biotechnology, Catholic University of BrasiliaBrasilia, Brazil

**Keywords:** proteins, peptides, anti-parasitic, host insects, tropical diseases

## Abstract

Africa, Asia, and Latin America are regions highly affected by endemic diseases, such as Leishmaniasis, Malaria, and Chagas’ disease. They are responsible for the death of 1000s of patients every year, as there is not yet a cure for them and the drugs used are inefficient against the pathogenic parasites. During the life cycle of some parasitic protozoa, insects become the most important host and disseminator of the diseases triggered by these microorganisms. As infected insects do not develop nocive symptoms, they can carry the parasites for long time inside their body, enabling their multiplication and life cycle completion. Eventually, parasites infect human beings after insect’s transmission through their saliva and/or feces. Hence, host insects and general arthropods, which developed a way to coexist with such parasites, are a promising source for the prospection of anti-parasitic compounds, as alternative methods for the treatment of protozoa-related diseases. Among the molecules already isolated and investigated, there are proteins and peptides with high activity against parasites, able to inhibit parasite activity in different stages of development. Although, studies are still taking their first steps, initial results show new perspectives on the treatment of parasitic diseases. Therefore, in this report, we describe about peptides from host insect sources with activity against the three most endemic parasites: *Leishmania* sp., *Plasmodium* sp., and *Trypanosome*s. Moreover, we discuss the future application insect peptides as anti-parasitic drugs and the use of non-hosts insect transcriptomes on the prospection of novel molecules for the treatment of parasitic neglected diseases.

## Introduction

In 1970, the Rockefeller Foundation introduced the term “the Great Neglected Diseases,” corresponding to several illnesses caused by infectious microorganisms and parasites (viruses, bacteria, fungi, protozoa, and helminthes), endemic in poor populations of Asia, Africa, and America. Neglected diseases received this name due to the lack of interest by health science researchers and pharmaceutical companies on developing and commercializing products for their treatment or cure. Consequently, studies on this field are poorly invested (Souza, 2010).

However, during the last 20 years, the pattern of neglected diseases worldwide has changed, as mortality rates are decreasing, while morbidity still grows on the population. All neglected diseases are closely related to poverty, lack of hygiene, food and quality of life. Therefore, it is difficult to include long-time treatment programs, as most of the patients have no financial conditions to pay for the drugs (Paes and Silva, 1999; Brasil, 2010).

Chagas diseases, Leishmaniasis, and Malaria are some of these neglected diseases. Chagas disease, first reported in America during 1909, was classified as an enzootic illness affecting mostly the poor population of Latin America. Nevertheless, nowadays, it became a worldwide concern, as it spread through many countries in Africa, Europe, and Asia (Coura and Viñas, 2010; World Health Organization [WHO], 2014, 2015a). Migration processes of contaminated people to non-endemic regions and environmental changes in forestry areas, favoring trypanosome parasites to adapt into domestic environment, increased the identification of Chagas disease cases in North America, Western Pacific region and at least 16 countries of Europe. Therefore, new forms of parasite transmission were described, including blood transfusion, organ transplantation, and vertical transmission from mother to child (Coura and Viñas, 2010; World Health Organization [WHO], 2014). The constant migration of people from different countries during the last two decades enlarged epidemiological and social matters, leading to an evaluation of economic and political aspects for the prevention, treatment, and eradication of this disease (World Health Organization [WHO], 2015a).

Leishmaniasis is another neglected disease with high concern to many countries worldwide. In February, the World Health Organization [WHO] (2015b) estimated 1.3 million new cases of Leishmaniasis (visceral and cutaneous) and 20,000–30,000 deaths annually. The cutaneous form of Leishmaniasis occurs in different populations of the world, although higher rates are observed in South American and African countries. On the other hand, 90% of all cases of visceral Leishmaniasis is concentrated in Bangladesh, Brazil, Ethiopia, India, Sudan, and South Sudan (Alvar et al., 2012). In Brazil, Leishmaniasis is showing an expansion, generally associated with environmental modification caused by man, population displacements arising from endemic areas and insufficient infrastructure in water and sewer systems (Paes and Silva, 1999; Brasil, 2010).

Additionally, Malaria is one of the most important parasitic infectious disease of the world. It occurs in tropical and subtropical areas of 106 countries worldwide. Only in 2012, 627,000 1000 people in the Saharan Africa died of Malaria (Center for Disease Control and Prevention [CDC], 2014). Although an estimative from the World Malaria Report consider a significant decrease in the number of countries exposed to Malaria (from 140 to 106), every year, 100s of millions of new episodes are registered in different regions of the world (Public Health England, 2014; World Malaria Report, 2015).

Most of neglected diseases have low financial support, both from Private and Governmental funds. However, great amount of international funding is given to control new cases of Malaria in endemic countries, as well as applied on preventive Programs for poor populations. The amount invested on the control of Malaria worldwide raised from US$ 960 million in 2005 to US$ 2.5 billion in 2014 (World Malaria Report, 2015). The number increased 8% between 2013 and 2014, but the financial support invested on the prevention and treatment of Malaria are still not sufficient to contain the arrival of new cases.

Hence, part of the financial investments applied on Malaria, Leishmaniasis, and Chagas’ disease is spent on research for the discovery of novel molecules that can be used as new pharmaceutical products for the treatment of these illnesses. Recently, studies demonstrated that the use of peptides isolated from host mosquitoes and other invertebrates, called anti-parasitic peptides (APPs), are the key to inhibit or even kill protozoa cells infecting the human body (Li et al., 2006; Moreira et al., 2007; Tian et al., 2008; Fieck et al., 2010; Gao et al., 2010; Rangel et al., 2011; Marr et al., 2012).

Therefore, this report describes the first studies related to the activity of different peptides from insect hosts APPs against three parasite species: *Trypanosoma* sp., *Leishmania* sp., and *Plasmodium* sp. The results reported here show the great potential of using insect host proteins and peptides to treat Chagas disease, Leishmaniasis, and Malaria in human patients.

## New Strategies for the Treatment of Chagas Disease

Chagas disease is a human trypanosomiasis endemic in Latin America countries, caused by the species *Trypanosoma cruzi*. The name was given by the scientist Carlos Chagas, in honor of the research developed by Oswaldo Cruz, during the beginning of the 20th century. The transmission occurs when a person is bitten by a triatomine insect containing *T. cruzi* parasites (Don and Chatelain, 2008). Common triatomines of Chagas disease vectors belong to *Triatoma, Rhodnius*, and *Panstrongylus generi.* In Central and South America, the most common triatomine is *T. prolixus* (Fieck et al., 2010).

Chagas’ disease can be classified into three different stages: acute, asymptomatic, and chronic. The acute stage consists of fever, facial edema, generalized lymphadenopathy, and hepatosplenomegaly. Usually, 5% of contaminated children die during this stage, but the illness can spontaneously undertake itself in 4–6 weeks, becoming asymptomatic. During this stage, also called indeterminate, the parasite passes to its second phase, which can last from 10 years to a lifetime. The last and most concerning stage is the chronic phase, once the disease can compromise the heart, leading to death by cardiac arrhythmia or congestive heart failure (Whitebread et al., 2005; Don and Chatelain, 2008). Therefore, Chagas disease has become one of the main causes of heart problems in Latin America countries, as well as gastrointestinal mega-syndromes in some patients (Chagas, 1981; Parada et al., 1997).

It is estimated that 11 million people in American countries are infected with *T. cruzi*, and 10–30% of them will develop the chronic stage of Chagas disease (Bern et al., 2007). There is no cure for Chagas disease and the available treatments correspond to the use of benznidazole or nifurtimox. Both are effective only in 50% of the cases, and for patients in the acute or early indeterminate stages. There is still no treatment, nor even a cure, for the late indeterminate and chronic stages of Chagas disease (Kennedy, 1997; Whitebread et al., 2005; Frearson et al., 2007).

Until now, there are also no vaccines developed to prevent infection by *T. cruzi*, neither effective vector eradication Programs to control parasite dissemination (Kevin, 2014). Thus, there is a strong need for the development of adequate therapeutic strategies to treat Chagas disease. Preliminary work described the use of peptides capable of lysing different cell types (Kehoe and Timmis, 1984). Therefore, the utilization of proteins and peptides from different sources became a strategic alternative for the control of parasites.

Another interesting feature is the inability of parasites to damage the cells of their insect hosts. Although, the infection with the parasite can cause several damages in human organs, it is harmless to triatomine insects (Rosendaal, 1997). In this way, investigating proteins and peptides from parasite’s insect hosts is a prospering alternative for the development of new tools against Chagas disease.

### Anti-trypanosomal Peptides

There are still, in literature, very few studies on the prospection and evaluation of the anti-trypanosomal effect of proteins and peptides from insect sources, especially for species causing Chagas disease. Among all classes of proteic molecules, only three have been studied for this purpose: apidaecins, cecropins, and melittins (**Table 1**).

**Table 1:** Insect peptides with anti-parasitic activity.

Anti-parasitic activity	Peptide	Insect species	Targeted parasite(s)	Half maximal inhibitory concentration (IC_50_)	Mortality rate	Reference
Anti-tripanosomal	Cecropin	*Hyalophora cecropia*	*Trypanosoma cruzi*	0.8 μM	NI	Fieck et al., 2010
	Melittin	*Apis melífera* venom	*T. cruzi*	0.8 μM	NI	Fieck et al., 2010
	Meucine-24	*A. melífera* venom	*T. cruzi*	10–20 μM	NI	Gao et al., 2010
Anti-malarial	Cecropin B	*H. cecropia*	*Plasmodium* sp.	0.5 μg/μl (128 μM)		Gwadz et al., 1989
	Cecropin A	*H. cecropia*	*P. falciparum*	>100 μM	NI	Boman et al., 1989
	Drosomycin 1	*Drosophila melanogaster*	*P. berghei*	NI	76% mortality at 20 μM	Tian et al., 2008
	Drosomycin 2	*D. melanogaster*	*P. berghei*	NI	29% mortality at 20 μM	Tian et al., 2008
	Gambicin	*Anopheles* sp.	*P. berghei*	10 μM	NI	Vizioli et al., 2001
	Gomesin	*A. gomesi*	*P. falciparum, P. berghei*	75.8–86.6 μM	NI	Moreira et al., 2007
	Meucine-25	*Mesobuthus eupeus*	*P. berghei*	10–20 μM	NI	Gao et al., 2010
	Scorpine	*Pandinus imperator*	*P. berghei*	NI	98% mortality at 15 μM	Carballar-Lejarazu et al., 2008
			*P. falciparum*	NI	100% mortality at 5 μM	Carballar-Lejarazu et al., 2008
Anti-leishmanial	Decoralin	*Oreumenes decoratus*	*L. major*	72 μM	NI	Konno et al., 2007
	Decoralin-NH2	Derived from Decoralin	*L. major*	11 μM	NI	Konno et al., 2007
	Eumenitin	*Eumenesru rubronotatus*	*Leishmania* sp.	35 μM	NI	Rangel et al., 2011
	Spinigerin	*P. messpiniger*	*L. donovani*	150 μM	NI	Landon et al., 2006
	Tachyplesin	*Tachypleus tridentatus*	*L. braziliensis*	12.5 μM	NI	Löfgren et al., 2008

*NI, not indicated by the authors.*

Apidaecins correspond to a group of small prolin-rich peptides, ranging from 18 to 20 amino acid residues, previously isolated from the honeybees *Apis melifera* (Casteels et al., 1989; Li et al., 2006). They have been widely studied as potential molecules for therapeutic use due to their anti-bacterial activity and harmless impact against human and animal cells (Casteels and Tempst, 1994; Li et al., 2006). Recent studies showed that this class of peptides can also present anti-trypanocidal activity at an absolute lethal concentration (LC_100_) of 199 μM (Fieck et al., 2010). Moreover, when used in pairwise treatments, together with magainin II (from the *Xenopus laevis*), cecropin (from the silk worm) and melittin (also from honeybee), the anti-trypanocidal activity increased 10 folds, showing additive effects at a half maximal inhibitory concentration (IC_50_) of 0.70 μM (Fieck et al., 2010).

Cecropins include a class of small and basic peptides of about 31–37 amino acid residues. They were first isolated from the silk moth *Hyalophora cecropia*, and have been described as an important molecule in the cell-free immunity of insects, with activity against bacteria and fungi growth (Boman, 1991; Boman et al., 1991). Earlier studies demonstrated the performance of synthetic cecropins against *T. cruzi.* The synthetic peptides, called SB-37 and Shiva-1, with punctual mutations in their amino acid sequence, were able to kill trypomastigote forms of the parasite after 1-h exposure, in a dose-responsive manner (Jaynes et al., 1988). Interestingly, Shiva-1 peptide was 10 times more effective against Trypomastigotes than SB-37, in terms of parasite damage and lysis.

Another report showed that a Cecropin A displayed trypanostatic effects against Chagas diseases parasite, inhibiting its growth at a LC_100_ of 80 μM, although it was not able to kill *T. Cruzi* cells (Fieck et al., 2010). However, when coupled with other peptides, such as magainin, an antagonistic effect was observed, at a concentration of 0.1 and 1.0 μM for cecropin and magainin, respectively (Fieck et al., 2010).

Furthermore, melittin is a small 26-amino acid peptide isolated from honeybee venom with anti-microbial activity against yeasts and bacteria (Lubke and Garon, 1997; Klotz et al., 2004; Lazarev et al., 2005). It also has showed ability to inhibit protein kinase C, protein kinase II, and myosin kinase (Yang and Carrasquer, 1997). Previous reports described the potential use of melittin on the treatment of epilepsy, HIV infection, and cancer (Loftus, 2009; Hood et al., 2013; Verma et al., 2013). Further studies demonstrated that melittin present inhibitory activity against trypomastigotes (LD_100_ = 30 μM). Also, when coupled with apidaecin, the parasite inhibitory activity was increased. Nevertheless, when melittin was coupled with cecropin, it showed an antagonistic effect, contrary to the additive effect demonstrated with apidaecin (Fieck et al., 2010).

## *Leishmania* sp.: A Mammalian Parasite to be Defeated

*Leishmania* is a genus of protozoa transmitted between mammals by blood-sucking sandflies. Mammal species, including dogs, mice and humans, are natural hosts for *Leishmania* and in some parts of the world, like India, humans are the main hosts (Kaye and Scott, 2011). The parasite’s live cycle encompasses a differentiation of procyclic promastigote form into infective metacyclic promastigote. This stage occurs inside sandflies, where the parasite will be ready for transmission at the stomodea valve’s insect. During blood-sucking, contaminated sandflies can inject metacyclic promastigotes into mammal tissues together with some parasite immunomodulatory elements. After phagocytized by host cells, parasites will accommodate inside human body. Then metacyclic promastigotes can become flagellate amastigote and replicates into host cells driving them to rupture, spreading amastigotes into host tissues and allowing infection of others phagocytes. The cycle is completed when infected phagocytes are sucked by another sand-fly and are converted in promastigotes into sand-fly’s midgut, re-starting a new cycle (Kaye and Scott, 2011).

There are more than 20 species of *Leishmania* related to several subtypes of chronic skin and viscera infections, classified as mucocutaneous disease, visceral disease, Leishmaniasis recidivism, and post-kala-azar dermal Leishmaniasis (McGwire and Satoskar, 2014). Around 1.3 million cases of Leishmaniasis are reported annually worldwide and there is an estimative that 350 million people in 88 countries are living at risk of developing Leishmaniasis (Kedzierski, 2010; World Health Organization [WHO], 2015b).

There is a huge number of drug treatment available to each form of Leishmaniasis. Pentavalent antimony is the agent of choice for the majority forms of such disease (Baiocco et al., 2009). Amphotericin B, Paromomycin, Pentamidine also have been used worldwide (Jaureguiberry et al., 2006; Chattopadhyay and Jafurulla, 2011; Sundar et al., 2011). However, these drugs are often harmful to the patient, the treatments need constant follow up and patients may not be 100% recovered (McGwire and Satoskar, 2014). Therefore, new strategies to treat Leishmaniasis is a clear need for millions of patients around the world.

### Anti-leishmanial Peptides

Some APPs were reported as acting against *Leishmania* species and have been considered a low harmful alternative, despite of current drugs used to control *Leishmania* infections (Chadbourne et al., 2011). Marr et al. (2012) listed some leishmanicidal peptides, such as Temporins, Bombins, Magainins, and Cathelicidins (from amphibians and mammalians), and discussed their mechanism of action. Arthropods Leishmanicidal peptides are described in **Table 1**.

Hence, among the APPs from insect sources, there is Spinigerin. It is a cysteine-free peptide constitutively expressed by termite *Pseudacanthotermes spiniger.* Its minimal inhibitory concentration (MIC) in bioassays against filamentous fungi, yeast, gram-negative bacteria is <5 μM (Landon et al., 2006). When assayed against *Leishmania donovani*, spinigerin was able to cause apoptosis-like cell death (IC_50_, 150 μM). Landon et al. (2006) suggested that such peptide could cause such effects by inhibiting some Leshimanial trypanothione reductase, thus, arresting detoxification processes against reactive oxygen species (ROS) produced by the host cells.

Eumenitin is a class of small cationic peptides (15 amino acids) from the venom of the solitary eumenine *Eumenes rubronotatus*, which was isolated and characterized by Konno et al. (2006). Its activity against Gram-positive and Gram-negative bacteria, as well as its capacity to stimulate degranulation of mast cells are known since its discovery. However, eumenitin’s leishimanicidal activity was reported only in Rangel et al. (2011), when the peptide was assayed against *Leishmania major*, displaying anti-parasitic effects at IC_50_ of 35 μM.

Decoralin belongs to a class of small and linear cationic α-helical peptides not stabilized by disulfide bonds. It was isolated for the first time in Konno et al. (2007) from the venom of the solitary eumenine wasp *Oreumenes decoratus*. Such class of peptides is known to adopt an amphipathic α-helical conformation, which was reported as essential for its biological activity (Powers and Hancock, 2003). Hence, decoralin showed activity against *L. major* at micromolar concentrations (IC_50__=_ 72 μM). But when the C-terminal of the peptide was amidated, the anti-parasitic activity demonstrated a IC_50_ sixfold lower, around 11 μM (Konno et al., 2007). Such enhance in its activity is speculated to be due a stabilization of α-helical conformation caused by C-terminal amidation, as well as an extra electrical charge provided by amidation (Sforça et al., 2004).

Tachyplesin is a class of anti-microbial and APPs from horseshoe-crab (*Tachypleus tridentatus*), described for the first time in Nakamura et al. (1988). This peptide is 17 amino acids long and have a beta conformation, been active against Gram-positive and negative bacteria, as well as against virus and cancer cells (Shen and Wu, 2014). Bioassays using *Leishmania braziliensis* demonstrated that tachyplesin could also inhibit this parasite’s growth at micromolar levels (12.5 μM; Löfgren et al., 2008).

Therefore, anti-leishmanicidal peptides from insect sources encompass a safe alternative to control *Leishmania* infection, once they are highly effective, but not as harmful as the current used drugs. The application of biotechnological tools for the large-scale production of such peptides are essential to aim this goal.

## Malaria: New Approaches for the Control of *Plasmodium* Species

Malaria remains one of the most prevalent and debilitating parasitic infection across Africa, Asia, and America continents. According to the World Health Organization (WHO), approximately 207 million people were diagnosed with Malaria and 627,000 died worldwide from it during 2012 (Ramasamy, 2014). Four species are the main cause of Malaria: *Plasmodium falciparum, P. vivax, P. malariae*, and *P. ovale*, transmitted by over 70 species of *Anopheles*’ mosquitoes. Among the parasites, *P. falciparum* is the most severe and responsible for 90% of all malaria deaths (Grimberg and Mehlotra, 2011).

*Plasmodium*’s life cycle is complex, involving multiple developing stages and locations, both in mosquitos and humans. Although its complex infectious cycle offers multiple sites for the development of specific drugs, finding a universal drug against malaria has been a challenging task. The major problem with the currently available anti-malarial drugs is the consistent resistance developed by some parasites, well-reported in literature (Bloland, 2001; White, 2004; Carter and Hurd, 2010; Torrent et al., 2012). Recently, the emerging resistance toward the drug artemisinin decreased even more the chances for a cure of Malaria, once that it used as first-line treatment for uncomplicated cases caused by *P. falciparum*, in most endemic countries (Biamonte et al., 2013).

Therefore, the demand for novel therapeutic agents against Malaria is urgent, especially using new targets. Nowadays, the new generation of strategies involves the blockage of malaria transmission by employing genetically modified vectors, mosquito pathogens or symbionts that express anti-parasite molecules (Carter et al., 2013).

Additionally, APPs from the innate immune system of insect vectors have been used as sources to control sporogonic stages of the malaria parasites. There are three sources of APPs that are active against sporogonic forms of parasites: (1) Endogenous – peptides that form part of the natural mosquito immune repertoire; (2) Exogenous – peptides isolated from other species; and (3) Synthetic – rational design of novel compounds (Carter and Hurd, 2010).

### Anti-malarial Peptides

As there is a bigger number of studies on Malaria, there are also more peptides presenting activity against *Plasmodium* species isolated from different arthropods (**Table 1**), including its insect host, *Anopheles* sp. (Gwadz et al., 1989; Vizioli et al., 2001; Kim et al., 2004; Bell, 2011).

Thus, during *Anopheles*’s *life* cycle, malaria mosquito vector (female anopheline) do not develop symptoms related to any infection. However, it was demonstrated that the insect produces three classes of anti-malarial peptides: cecropins, defensins, and gambicins. Recent reports showed that the three classes of peptides present activity against mosquito-stage and, in some cases, blood stage parasites (Bell, 2011). Thus, cecropins A and B demonstrated activity against ookinetes and oocysts of *P. falciparum, P. berghei, P. knowlesia*, and *P. cymonolgi* (Gwadz et al., 1989; Kim et al., 2004), while gambicin displayed 54–64% lethality against *P. berghei* ookinetes at 10–100 μM (Vizioli et al., 2001).

The first report of APPs was described in Boman et al. (1989). In this study, they analyzed the growth inhibition of *P. falciparum* cells using different concentrations of cecropin and melittin peptides, called CA (l–13) and H (l–13; Boman et al., 1989). During the same year, Gwadz et al. (1989) demonstrated the anti-malarial activity of Cecropin B (from giant silk moths) at a concentration of 0.5 μg/μl (128 μM). The peptide prevented normal development of oocysts from *Plasmodium* species in *Anopheles gambiae* insects previously infected with the parasite (Gwadz et al., 1989).

Other important source of anti-malarial peptides was isolated from scorpion and spiders’ venoms. Among these peptides, there is scorpine, isolated from the scorpion *Pandinus imperator*, whose structure resembles a hybrid between a defensin and a cecropin. This peptide was responsible for the mortality of 98% of *P. berghei* gametocytes at 15 μM and 100% reduction of *P. falciparum* parasitemia at 5 μM (Carballar-Lejarazu et al., 2008).

Meucine-24, a α-helical peptide with an N-terminal homologous to melittin and meucine-25 have been isolated from the venom of the scorpion *Mesobuthus eupeus* and showed ability to inhibit the development of *P. berghei* ookinetes at micromolar concentrations (IC_50_ 10–20 μM; Gao et al., 2010).

Moreover, several insect defensins have displayed anti-malarial activity. Among them, there are two drosomycins isolated from the *Drosophila melanogaster* hemolymph. Drosomycin1 is able to inhibit the development of *P. berghei* ookinetes in 76% at 20 μM, while Drosomycin 2 is able to retard the development of the same parasite in 29% at 20 μM. (Tian et al., 2008). Similarly, gomesin, an insect defensin isolated from the hemocytes of the spider *Acanthoscurria gomesiana*, inhibited the *in vitro* growth of intra-erythrocytic forms of *P. falciparum*, causing a dramatic reduction of gametocyte exflagelation and oocyst population in both *P. falciparum* and *P. berghei* (Moreira et al., 2007).

As described here, multiple compounds can be pharmaceutically attractive to treat Malaria, once that current available anti-malarial drugs are inefficient against parasites. Therefore, novel peptides can open new perspectives for the development of new anti-parasitic drugs (Leavy, 2010; Vale et al., 2014).

### Structure and Mode of Action of Anti-malarial Peptides

The description of several anti-microbial peptides with activity against pathogenic bacteria, fungi, protozoa and other harmful organisms are well-described in literature. Those peptides are isolated from different sources, varying from human beings, to plants, insects, marine animals, and even microorganisms (Pelegrini et al., 2007; Lacerda et al., 2014; Vale et al., 2014; Kang et al., 2015). However, few of them show anti-parasitic activity, especially related to human neglected diseases (Li et al., 2006; Moreira et al., 2007; Tian et al., 2008; Fieck et al., 2010; Gao et al., 2010; Rangel et al., 2011; Marr et al., 2012). Among these diseases, Malaria has the most number of published articles on the discovery and characterization of anti-plasmodium proteins and peptides. Considering insect peptides with anti-parasitic activity, Malaria continues reaching the first position in tested molecules, although there are still very few studies demonstrating anti-malarial activities of insect peptides for future use as drugs (Boman et al., 1989; Gwadz et al., 1989; Vizioli et al., 2001; Kim et al., 2004; Moreira et al., 2007; Carballar-Lejarazu et al., 2008; Tian et al., 2008; Gao et al., 2010; Vale et al., 2014).

Recently, anti-malarial peptides from insect sources were classified into four groups: (i) α-helical peptides deprived of Cys residues; (ii) β-pleated peptides containing disulfide bridges; (iii) peptides rich in Pro, Gly, His, Arg, and Trp residues; and (iv) circular anti-microbial peptides (Vale et al., 2014). Out of these groups, only the first one contains peptides from insect sources with anti-parasitic activities against *Plasmodium* species.

Thus, α-helical peptides deprived of Cys residues correspond to linear cationic small peptides with α-helical structure, whose positive charges come from the presence of Lys and Arg residues. Another feature is the existence of at least 50% of their content composed by hydrophobic amino acid residues (Powers and Hancock, 2003). Cecropins (from *H. cecropia*) and Melittins (from *A. mellifera* venom) are some examples of α-helical peptides deprived of Cys residues, due to their α-helical domains and content of hydrophobic residues in the amino-terminal site, respectively (Raghuraman and Chattopadhyay, 2007; Tanaka et al., 2008).

**FIGURE 1:**
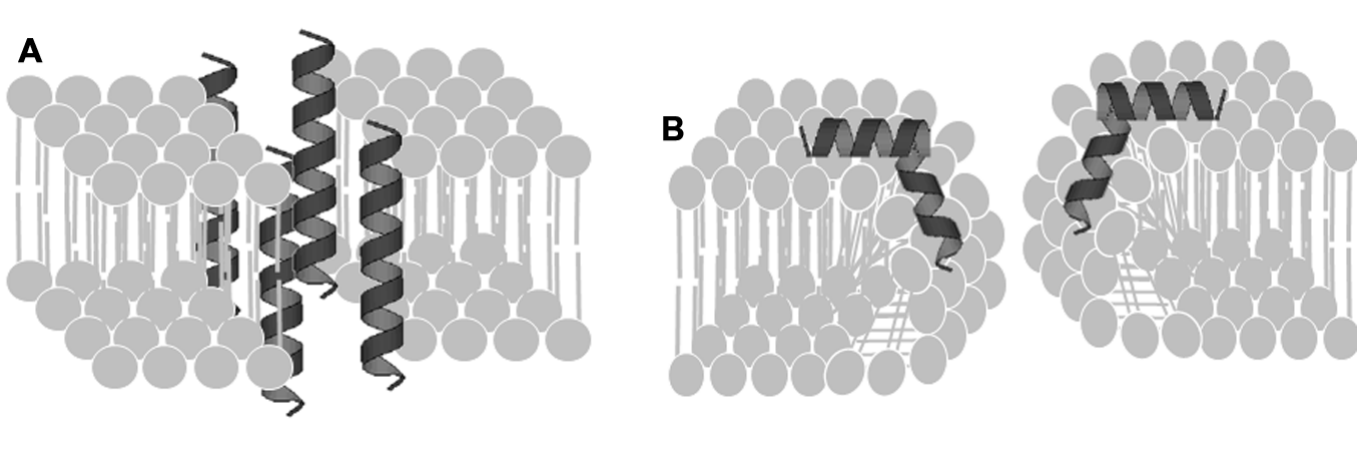
**Mechanism of action of anti-malarial peptides. (A)** Ordered toroidal pore formation. **(B)** Disoredered toroidal pore formation (Source: Segunpta et al., 2008 with modifications).

For insect α-helical peptides deprived of Cys residues, the mode of action consists of poration of the lipid membrane through a disordered toroidally shape lining of peptides (**Figure 1**; Yang et al., 2001; Segunpta et al., 2008). The charged amino acid residues showed to be essential for electrostatic interactions with the lipid membrane, resulting in pore formation (Segunpta et al., 2008).

In general, the mechanism of action of anti-malarial peptides from insect sources are still poorly studied. However, the activities of distinct APPs obey many of the rules governing their ability to disrupt bacterial membranes. The interaction with APPs to the pathogen’s cell membrane appears to be acutely influenced by the respective charges and amphipathies of the reactants. In fact, a comparison between the peptides’ ability to inhibit the growth of malaria parasites and that of bacteria demonstrates a remarkable parallelism in the way that each modification affects both activities (Mor, 2009).

## Transcriptome: Prospection of Novel Molecules for the Control of Parasitic Diseases

Transcriptome studies are important to understand the physiology and mechanism of action of parasitic molecules and have been one of the alternatives in seeking resolutions to certain diseases, such as Malaria (Tarun et al., 2008). In this report, transcriptome and proteome of malaria parasite liver stages displayed essential knowledge about how the parasite behaves inside host cells. Moreover, transcriptome studies are also being used to better understand parasite gene profile during infection, as demonstrated by Ngwa et al. (2013). The report showed changes in *Plasmodium falciparum* transcriptome pattern during its initial phase, describing that modifications were observed even during the first half hour after human transmission by the mosquito (Ngwa et al., 2013). Similarly, Hellgren et al. (2013) used transcriptome data from birds infected with *P. relictum*, which causes avian Malaria, to identify and characterize the MSP1 gene (SGS1 and GRW4). The analysis of those genes could led to the generation of a new vaccine for the prevention of avian Malaria, also becoming a relevant result for epidemiological studies (Hellgren et al., 2013).

The evaluation of insect transcriptomes, especially the ones that are hosts of human parasites, have been published for some species. Hence, analyses of *Musca domestica* transcriptome revealed new insights and genes involved in the prophenoloxidase system (proPO), a pathway related to melanization in arthropods. The melanin production occurs after infection, promoting protection against invasion of pathogens, such as bacteria (Li et al., 2015). Therefore, the novel information obtained from transcriptome studies allowed a better understanding on the mechanism of action and immune functions of the proPO system in *M. domestica*.

The inefficiency of therapeutic strategies to treat protozoa-related diseases and the frequent incidence of resistance developed by parasites led to the search for molecules that can be used as new pharmaceutical products for the treatment of parasitic diseases. In the last years, transcriptomes have been employed as promissory source for the prospection of active proteins and peptides. The availability of high-throughput transcriptomic technologies has contributed to considerably enhance the knowledge to understand host–parasite interactions and to identify new and effective treatments for parasitic diseases, especially neglected ones (Cantacessi et al., 2015; Davies et al., 2015). Furthermore, even transcriptome of phytopathogenic insects could be an interesting source for future prospections of proteins and peptides for therapeutic use on the treatment of Chagas disease, Leishmaniasis, and Malaria (Firmino et al., 2013; Fonseca et al., 2015).

## Anti-Parasitic Peptides: Future Reality for Drug Development?

Several researches are published every year on the prospection and discovery of new molecules from different sources with high potential use as drugs against bacterial, fungi, and parasitic infections (Lacerda et al., 2014; Breen et al., 2015; Kang et al., 2015; Tam et al., 2015). Insect peptides with anti-parasitic activity demonstrate to be an interesting tool on the treatment of neglected diseases, such as Malaria, Leishmaniasis, and Chagas disease.

Many reasons explain why neglected diseases are out of reach from eradication. Hygiene issues, social and economic status of the population and endemic areas full of viable parasite vectors are some of them (Alvar et al., 2006; European Alliance Against Malaria, 2007; Klein et al., 2012). Nevertheless, drug inefficiency and misuse, and serious side effects are the main causes of why these diseases are still increasing in number and area worldwide.

The mechanism of action of the drugs used nowadays for the treatment of some neglected diseases are yet not well-known, facilitating mutations triggering drug resistance by the parasites. Moreover, the poor efficient response of such drugs, combined with strong side effects, stimulates the development of alternatives for a better treatment of neglected diseases (Teixeira et al., 2014; Vale et al., 2014; Visser et al., 2014). As it is occurring with anti-bacterial drugs, the misuse of anti-parasitic drugs is also leading to resistance, increasing preoccupation about a growing arrival of new cases and inefficacy on the treatment of recurrent cases (White, 2004; Dondorp et al., 2009; Chakravarty and Sundar, 2010; Alsford et al., 2012; Whyllie et al., 2015).

Nowadays, anti-parasitic drug candidates containing peptides are still very few, with most of them at Clinical Trials stage (Fox, 2013). Nonetheless, the fact that pharmaceutical companies continue avoiding the application of peptides for the production of drugs goes beyond oral bioavailability and drug resistance issues. Peptide degradation by digestive enzymes and interactions with plasma proteins are some conditions that complicate the utilization of APPs for production of drugs. Furthermore, the cost of scaling-up the production of organic compounds is still high, contributing to expensive drugs into the market (Fox, 2013; Vale et al., 2014).

However, despite all disadvantages, since the 1990s, the scenario of drug production is changing. The advances on biotechnology applied for the development of new processes for the production of proteins and peptides for therapeutic use stimulated the formulation of different drugs with high specificity and activity toward their targets, at lower costs and using faster production methods (Leavy, 2010). Novel improvements on biocompatible carriers to extend peptide biocompatibility also contributed for enhancing target reach of peptide’s site of action (Costa et al., 2011; Maia et al., 2014). Similarly, use of nanotechnology and peptide encapsulation using biodegradable polymers allowed to increase the precision of drug target, as well as enabled long-acting release forms of peptides in the organism (Anthony and Freda, 2009; Stark, 2011). Therefore, by 2012, numerous peptide drugs were already approved in the US, reflecting in a market of 13 billion dollars, represented by 1.5% of total global drugs sales. Other countries also approved and released peptide drugs over the years, especially the Europeans Germany and UK, responsible for 63% of peptide therapeutics in the market, followed by France, Italy, Scandinavia, and Spain (Kaspar and Reichert, 2013; Vale et al., 2014).

Hence, although pharma companies’ main target were small molecules (below 500 Da), due to their ease of production and availability for oral application, small drug candidates showed low specificity for their targets, enhancing susceptibility to metabolic inactivation, which increase side effects, dose-response, and decrease their bioavailability (Craik et al., 2013). Peptides with average size of 5 kDa, however, displayed high specificity toward their targets, decreasing not only the dose of use, but also harmful side effects. For example, peptide drugs, such as NVB302, OP-145, Omiganan and Pexiganan, are now in Clinical Trials (Phases I, II, and III, respectively), as future effective alternatives for the treatment of Malaria (Fox, 2013). Therefore, peptide drugs for the treatment of neglected diseases is a reality for future years, with high chances of improvement and expanding in terms of variety and application.

## Conclusion

As described before in this report, the activity of insect peptides against the parasites *Trypanosoma* sp., *Leishmania* sp., and *Plasmodium* sp. encourages advances in this research investigation, once there is still a great necessity on developing new effective drugs for the treatment of Chagas disease, Leishmaniasis, and Malaria, respectively. Although poorly explored, insect peptides demonstrate high potential for future application in therapeutics of infectious diseases, especially parasitic neglected ones.

Currently, Malaria, together with other infection disease, such as Tuberculosis and AIDS, receives annually significant amounts of international resources for research on the development of new strategies for their treatment and prevention. Therefore, it was recently removed from the “Neglected disease” group. Nevertheless, there is still no treatment 100% efficient for this disease, but only methods to prolong the life and decrease the pain of patients (Olliaro et al., 2012; Uniting to Combat NTDs, 2014).

Similarly, Chagas disease continues to represent the parasitic illness responsible for the highest number of deaths in Latin America, surpassing Malaria. Considering that 23% of all infected patients with *T. cruzi* are located in Brazil, this country stands out in the number of published articles about the disease, the parasite, its host vector and on the discovery of novel tools to treat Chagas’s disease (more than 1400 publications during the last 70 years). Out of 55 research institutes working with Chagas’s disease worldwide, six are locate in Brazil. However, yet, there are very few effective and low toxic drugs available for its treatment.

In the absence of appropriate drugs, Leishmaniasis has also represented a health concern, due to its geographic expansion and urbanization trend. Moreover, the control of human *Leishmania* cannot be done without understanding canine Leishmaniasis, as pet dogs hosting parasites can easily transmit the disease to humans (Papadopoulou et al., 2005).

Advances on molecular biotechnology allow the production of different proteins and peptides in their active form using several heterologous systems. Therefore, scaling up the production of insect peptides for the development of pharmaceutical drugs to be used on the treatment of parasitic diseases has become a routine technique. Similarly, transcriptome analyses can be a relevant tool on the discovery of novel peptides from insect species with activity against parasites. Therefore, it is clear that insect peptides present high potential to become the future drugs for decreasing the number of infectious diseases, such as Malaria, Leishmaniasis, and Chagas disease.

It is not expected that biotechnological peptides will take over chemical molecules on the development of new drugs. However, peptides are already becoming new options for the treatment of several diseases, from oncology to infectious ones, as they can cause synergic effects, enhancing the efficacy of drugs at lower concentration. Therefore, peptide drugs have reached a place of interest by pharmaceutical companies, where despite of their cost of production and biochemical challenges to overcome, peptides demonstrate to be unique in their mechanism of action and therapeutic use.

Peptide drugs for neglected diseases, such as Malaria, Leishmaniasis, and Chagas diseases, are still at early stages compared to other drugs developed for chronic and non-infectious diseases (Lax, 2010). But even at a slow pace, studies and researches on peptide drugs are demonstrating to be a reality for the treatment of neglected diseases. For the next 10–15 years, new peptide drugs are expected to be at the market for the treatment of Malaria and, hopefully, for other neglected diseases including Chagas and Leishmaniasis.

## Author Contributions

AL: contributed with informations about anti-malarial peptides, introduction of the text, revision, and edition of the entire manuscript. PP: contributed with informations about peptides against Chagas disease’s peptides, introduction of the text, revision, and edition of the entire manuscript. EV: contributed with informations about anti-Leishmanial peptides, conclusion, and English revision of the manuscript. DdO: contributed with informations about transcriptome studies in the manuscript. MG-d-S: contributed with English and content revision of the manuscript.

## Conflict of Interest Statement

The authors declare that the research was conducted in the absence of any commercial or financial relationships that could be construed as a potential conflict of interest.

## References

[B1] Alsford S. Eckert S. Baker N. Glover L. Sanchez-Flores A. Leung K. F. (2012). High-throughput decoding of antitrypanosomal drug efficacy and resistance. *Nature* 25 232–236. 10.1038/nature10771 22278056 PMC3303116

[B2] Alvar J. Vélez I. D. Bern C. Herrero M. Desjeux P. Cano J. (2012). Leishmaniasis worldwide and global estimates of tis incidence. *PLoS ONE* 7:e35671 10.1371/journal.pone.0035671 PMC3365071 22693548

[B3] Alvar J. Yactayo S. Bern C. (2006). Leishmaniasis and poverty. *Trends Parasitol.* 22 552–557. 10.1016/j.pt.2006.09.004 17023215

[B4] Anthony L. Freda P. U. (2009). From somatostatin to octreotide LAR: evolution of a somatostatin analogue. *Curr. Med. Res. Opin.* 25 2989–2999. 10.1185/03007990903328959 19842996 PMC3678951

[B5] Baiocco P. Colotti G. Franceschini S. Ilari A. (2009). Molecular basis of antimony treatment in leishmaniasis. *J. Med. Chem.* 52 2603–2612. 10.1021/jm900185q 19317451

[B6] Bell A. (2011). Antimalarial peptides: the long and the short of it. *Curr. Pharm. Des.* 17 2719–2731. 10.2174/138161211797416057 21728986

[B7] Bern C. Montgomery S. P. Herwaldt B. L. Rassi A. Jr. Marin-Neto J. A. Dantas R. O. (2007). Evaluation and treatment of Chagas disease in the United States: a systematic review. *JAMA* 298 2171–2181. 10.1001/jama.298.18.2171 18000201

[B8] Biamonte M. A. Wanner J. Le Roch K. G. (2013). Recent advances in malaria drug discovery. *Bioorg. Med. Chem. Lett.* 23 2829–2843. 10.1016/j.bmcl.2013.03.067 23587422 PMC3762334

[B9] Bloland P. B. (2001). *Drug Resistance in Malarial.* Genevra: World Health Organization 32.

[B10] Boman H. G. (1991). Antibacterial peptides: key components needed in immunity. *Cell* 65 205–207. 10.1016/0092-8674(91)90154-Q 2015623

[B11] Boman H. G. Faye I. Lee J. Y. Gudmundsson G. H. Lidholm D. A. (1991). Cell-free immunity in *Cecropia*. A model system for antibacterial proteins. *Eur. J. Biochem.* 201 23–31. 10.1111/j.1432-1033.1991.tb16252.x 1915368

[B12] Boman H. G. Wadde D. Boman I. A. Wihlint B. Merrifield R. B. (1989). Antibacterial and antimalarial properties of peptides that are cecropin-melittin hybrids. *FEBS Lett.* 259 103–106. 10.1016/0014-5793(89)81505-4 2689223

[B13] Brasil (2010). *Doenças Infecciosas e Parasitárias: Guia de Bolso* 8 Rev Edn Brasília: Ministério da Saúde, Secretaria de Vigilância em saúde, Departamento de Vigilância Epidemiológica 444.

[B14] Breen S. Solomon P. S. Bedon F. Vincent D. (2015). Surveying the potential of secreted antimicrobial peptides to enhance plant disease resistance. *Front. Plant Sci.* 6:900 10.3389/fpls.2015.00900 PMC4621407 26579150

[B15] Cantacessi C. Dantas-Torres F. Nolan M. J. Otranto D. (2015). The past, present, and future of *Leishmania* genomics and transcriptomics. *Trends Parasitol.* 31 100–108. 10.1016/j.pt.2014.12.012 25638444 PMC4356521

[B16] Carballar-Lejarazu R. Rodriguez M. H. Hernnedez-Hernnedez F. C. Ramos-Castañeda J. Possanic L. D. Zurita-Ortega M. (2008). Recombinant scorpine: multifunctional antimicrobial peptides with activity against different pathogens. *Cell Mol. Life Sci.* 65 3081–3092. 10.1007/s00018-008-8250-8 18726072 PMC11131772

[B17] Carter V. Hurd H. (2010). Choosing anti-*Plasmodium* molecules for genetically modifying mosquitoes: focus on peptides. *Trends Parasitol.* 26 582–590. 10.1016/j.pt.2010.07.005 20800543

[B18] Carter V. Underhill A. Baber I. Sylla L. Baby M. Larget-Thiery I. (2013). Killer bee molecules: antimicrobial peptides as effector molecules to target sporonogic stages of *Plasmodium*. *PLoS Pathog.* 9:e1003790 10.1371/journal.ppat.1003790 PMC3836994 24278025

[B19] Casteels P. Ampe C. Jacobs F. Vaeck M. Tempst P. (1989). Apidaecins: antibacterial peptides from honeybees. *EMBO J.* 8 2387–2391. 2676519 10.1002/j.1460-2075.1989.tb08368.x PMC401180

[B20] Casteels P. Tempst P. (1994). *Apidaecin*-type peptide antibiotics function through a non-poreforming mechanism involving stereospecificity. *Biochem. Biophys. Res. Commun.* 199 339–345. 10.1006/bbrc.1994.1234 8123032

[B21] Center for Disease Control and Prevention [CDC] (2014). *Malaria Worldwide.* Available at: http://www.cdc.gov/malaria/malaria_worldwide/ [accessed October 20, 2014].

[B22] Chadbourne F. L. Raleigh C. Ali H. Z. Dennya P. W. Cobb S. L. (2011). Studies on the antileishmanial properties of the antimicrobial peptides temporin A, B and 1Sa. *J. Pept. Sci.* 17 751–755. 10.1002/psc.1398 21805542

[B23] Chagas C. (1981). *Carlos Chagas: Coletanea de Trabalhos Científicos* Vol. 6 Brasilia: Editora Universidade de Brasília 247–258.

[B24] Chakravarty J. Sundar S. (2010). Drug resistance in Leishmaniasis. *J. Glob. Infect Dis.* 2 167–176. 10.4103/0974-777X.62887 20606973 PMC2889657

[B25] Chattopadhyay A. Jafurulla M. (2011). A novel mechanism for an old drug: amphotericin B in the treatment of visceral leishmaniasis. *Biochem. Biophys. Res. Commun.* 416 7–12. 10.1016/j.bbrc.2011.11.023 22100811

[B26] Costa F. Carvalho I. F. Montelaro R. C. Gomes P. Martins M. C. (2011). Covalent immobilization of antimicrobial peptides (AMPs) onto biomaterial surfaces. *Acta Biomater.* 7 1431–1440. 10.1016/j.actbio.2010.11.005 21056701

[B27] Coura J. R. Viñas P. A. (2010). Chagas disease: a new worldwide challenge. *Nature* 56 S6–S7. 10.1038/nature09221 20571554

[B28] Craik D. J. Fairlie D. P. Liras S. Price D. (2013). The future of peptide-based drugs. *Chem. Biol. Drug Des.* 81 136–147. 10.1111/cbdd.12055 23253135

[B29] Davies P. D. Bodmer J. L. Felgner P. L. Doolan D. L. (2015). Large screen approaches to identify novel malaria vaccine candidates. *Vaccine* 33 7496–7505. 10.1016/j.vaccine.2015.09.059 26428458 PMC4729565

[B30] Don R. Chatelain E. (2008). “Drug discovery for neglected diseases: view of a public–private partnership,” in *Antiparasitc and Antibacterial Drug Discovery: From Molecules Targets to Drug Candidates* ed. Selzer P. M. (Blackwell: Wiley) 58–67.

[B31] Dondorp A. M. Nosten F. Yi P. Das D. Phyo A. P. Tarning J. (2009). Artemisinin resistance in *Plasmodium falciparum* malaria. *N. Engl. J. Med.* 361 455–467. 10.1056/NEJMoa0808859 19641202 PMC3495232

[B32] European Alliance Against Malaria (2007). *Malaria & Poverty.* London: European Alliance Against Malaria 1–4.

[B33] Fieck A. Hurwitz I. Kang A. S. Durvasula R. (2010). *Trypanosoma cruzi*: synergistic cytotoxicity of multiple amphipathic anti-microbial peptides to *T. cruzi* and potential bacterial hosts. *Exp. Parasitol.* 125 342–347. 10.1016/j.exppara.2010.02.016 20206169 PMC2875304

[B34] Firmino A. A. Fonseca F. C. de Macedo L. L. Coelho R. R. Antonino de Souza J. D. Jr. Togawa R. C. (2013). Transcriptome analysis in cotton boll weevil (*Anthonomus grandis*) and RNA interference in insect pests. *PLoS ONE* 8:e85079 10.1371/journal.pone.0085079 PMC3874031 24386449

[B35] Fonseca F. C. Firmino A. A. P. Macedo L. L. Coelho R. R. de Souza Júnior J. D. Silva-Junior O B. (2015). Sugarcane giant borer transcriptome analysis and identification of genes related to digestion. *PLoS ONE* 10:e0118231 10.1371/journal.pone.0118231 PMC4338194 25706301

[B36] Fox J. L. (2013). Antimicrobial peptides stage a comeback. *Nat. Biotechnol.* 31 379–382. 10.1038/nbt.2572 23657384

[B37] Frearson J. A. Wyatt P. G. Gilbert I. H. Fairlamb A. H. (2007). Target assessment for antiparasitic drug discovery. *Trends Parasitol.* 23 589–595. 10.1016/j.pt.2007.08.019 17962072 PMC2979298

[B38] Gao B. Xu J. Rodriguez Mdel C. Lanz-Mendonza H. Hernández-Rivas R. Du W. (2010). Characterization of two linear cationic antimalarial peptides in the scorpion *Mesobuthus eupeus*. *Biochimie* 92 350–359. 10.1016/j.biochi.2010.01.011 20097251

[B39] Grimberg B. T. Mehlotra R. K. (2011). Expanding the antimalarial drug arsenal – now but how? *Pharmaceuticals* 4 681–712. 10.3390/ph4050681 21625331 PMC3102560

[B40] Gwadz R. W. Kaslow D. Lee J. Y. Maloy W. L. Zasloff M. Miller L. H. (1989). Effects of magainins and cecropins on the sporogonic development of malaria parasites in mosquitoes. *Infect. Immun.* 57 2628–2633. 2759705 10.1128/iai.57.9.2628-2633.1989 PMC313504

[B41] Hellgren O. Kutzer M. Bensch S. Valkiunas G. Palinauskas V. (2013). Identification and characterization of the merozoite surface protein 1 (msp1) gene in a host-generalist avian malaria parasite, *Plasmodium relictum* (lineages SGS1 and GRW4) with the use of blood transcriptome. *Malar J.* 12 381 10.1186/1475-2875-12-381 PMC3827925 24172200

[B42] Hood J. L. Jallouk A. P. Campbell N. Ratner L. Wickline S. A. (2013). Cytolytic nanoparticles attenuate HIV-1 infectivity. *Antivir. Ther.* 18 95–103. 10.3851/IMP2346 22954649

[B43] Jaureguiberry P. Graby G. Caumes E. (2006). Efficacy of short-course intramuscular pentamidine isethionate treatment on old world localized cutaneous leishmaniasis in 2 patients. *Clin. Infect. Dis.* 42 1812–1813. 10.1086/504430 16705598

[B44] Jaynes J. M. Burton C. A. Barr S. B. Jeffers G. W. Julian G. R. White K. L. (1988). In vitro cytocidal effect of novel lytic peptides on *Plasmodium falciparum* and *Trypanosoma cruzi*. *FASEB J.* 2 2878–2883. 3049204 10.1096/fasebj.2.13.3049204

[B45] Kang H. K. Seo C. H. Park Y. (2015). Marine peptides and their anti-infective activities. *Mar. Drugs* 13 618–654. 10.3390/md13010618 25603351 PMC4306955

[B46] Kaspar A. A. Reichert J. M. (2013). Future directions for peptide therapeutics developments. *Drug Discov. Today* 18 807–817. 10.1016/j.drudis.2013.05.011 23726889

[B47] Kaye P. Scott P. (2011). Leishmaniasis: complexity at the host–pathogen interface. *Nat. Rev.* 9 604–615. 10.1038/nrmicro2608 21747391

[B48] Kedzierski L. (2010). Leishmaniasis vaccine: where are we today? *J. Glob. Infect. Dis.* 2 177–185. 10.4103/0974-777X.62881 20606974 PMC2889658

[B49] Kehoe M. Timmis K. N. (1984). Cloning and expression in *Escherichia coli* of the streptolysin O determinant from *Streptococcus pyogenes*: characterization of the cloned streptolysin O determinant and demonstration of the absence of substantial homology with determinants of other thiol-activated toxins. *Infect. Immun.* 43 804–810. 6321351 10.1128/iai.43.3.804-810.1984 PMC264252

[B50] Kennedy T. (1997). Managing the drug discovery/development interface. *Drug Discov. Today* 2 436–444. 10.1016/S1359-6446(97)01099-4

[B51] Kevin M. B. (2014). Chagas disease in the 21th century: a public health success or an emerging threat? *Parasite* 21:11 10.1051/parasite/2014012 PMC3952655 24626257

[B52] Kim W. Koo H. Richman A. M. Seeley D. Vizioli J. Klocko A. D. (2004). Ectopic expression of a cecropin transgene in the human malaria vector mosquito *Anopheles gambiae* (Diptera: Culicidae): effects on susceptibility to *Plasmodium*. *J. Med. Entomol.* 41 447–455. 10.1603/0022-2585-41.3.447 15185949

[B53] Klein N. Hurwitz I. Durvasula R. (2012). Globalization of chagas disease: a growing concern in nonendemic countries. *Epidemiol. Res. Int.* 2012:13 10.1155/2012/136793

[B54] Klotz S. A. Gaur N. K. Rauceo J. Lake D. F. Park Y. Hahm K. S. (2004). Inhibition of adherence and killing of *Candida albicans* with a 23-Mer peptide (Fn/23) with dual antifungal properties. *Antimicrob. Agents Chemother.* 48 4337–4341. 10.1128/AAC.48.11.4337-4341.2004 15504862 PMC525394

[B55] Konno K. Hisada M. Naoki H. Itagaki Y. Fontana T. Rangel M. (2006). Eumenitin, a novel antimicrobial peptide from the venom of the solitary eumenine wasp *Eumenesru bronotatus*. *Peptides* 27 2624–2631. 10.1016/j.peptides.2006.04.013 16762455

[B56] Konno K. Rangel M. Oliveira J. S. Cabrera M. P. S. Fontana R. Hirata I. Y. (2007). Decoralin, a novel linear cationic a-helical peptide from the venom of the solitary eumenine wasp *Oreumenes decoratus*. *Peptides* 28 2320–2327. 10.1016/j.peptides.2007.09.017 17981364

[B57] Lacerda A. F. Vasconcelos E. A. R. Pelegrini P. B. Grossi-de-Sa M. F. (2014). Antifungal defensins and their role in plant defense. *Front. Microbiol.* 5:116 10.3389/fmicb.2014.00116 PMC3980092 24765086

[B58] Landon C. Meudal H. Boulanger N. Bulet P. Vovelle F. (2006). Solution structures of stomoxyn and spinigerin, two insect antimicrobial peptides with anα-helical conformation. *Biopolymers* 81 92–103. 10.1002/bip.20370 16170803

[B59] Lax R. (2010). *The Future of Peptide Development in the Pharmaceutical Industry, Pharmanuacturing: the International Peptide Review.* Available at: http://www.polypeptide.com/web/upload/medias/1401702726538c49464a6f5.pdf [accessed December 17, 2015].

[B60] Lazarev V. N. Shkarupeta M. M. Titova G. A. Kostrjukova E. S. Akopian T. A. Govorun V. M. (2005). Effect of induced expression of an antimicrobial peptide melittinon *Chlamydia trachomatis* and *Mycoplasma hominis* infections in vivo. *Biochem. Biophys. Res. Commun.* 338 946–950. 10.1016/j.bbrc.2005.10.028 16246304

[B61] Leavy O. (2010). Therapeutic antibodies: past, present and future. *Nat. Rev. Immunol.* 10:297 10.1038/nri2763 20422787

[B62] Li D. Liang Y. Wang X. Wang L. Qi M. Yu Y. (2015). Transcriptomic analysis of musca domestica to reveal key genes of the prophenoloxidase-activating system. *Gen. Immun.* 5 1827–1841. 10.1534/g3.115.016899 PMC4555219 26156588

[B63] Li W. F. Ma G. X. Zhou X. X. (2006). Apidaecin-type peptides: biodiversity, structure-function relationships and mode of action. *Peptides* 27 2350–2359. 10.1016/j.peptides.2006.03.016 16675061

[B64] Löfgren S. E. Miletti L. C. Steindel M. Bachère E. Barracco M. A. (2008). Trypanocidal and leishmanicidal activities of different antimicrobial peptides (AMPs) isolated from aquatic animals. *Exp. Parasitol.* 118 197–202. 10.1016/j.exppara.2007.07.011 17888907

[B65] Loftus P. (2009). The buzz: targeting cancer with bee venom. *Wall Street J.* Available at: http://www.wsj.com/articles/SB10001424052970203803904574433382922095534 [accessed July 17, 2015].

[B66] Lubke L. L. Garon C. F. (1997). The antimicrobial agent melittin exhibits powerful in vitro inhibitory effects on the Lyme disease spirochete. *Clin. Infect. Dis.* 25 S48–S51. 10.1086/516165 9233664

[B67] Maia F. R. Barbosa M. Gomes D. B. vale N. Granja P. Gomes P. (2014). Hydrogel depots for local co-delivery of osteoinductive peptides and mesenchymal stem cells. *J. Control. Release* 189 158–168. 10.1016/j.jconrel.2014.06.030 24979208

[B68] Marr A. K. McGwire B. S. McMaster W. R. (2012). Modes of action of Leishmanicidal antimicrobial peptides. *Future Microbiol.* 7 1047–1059. 10.2217/fmb.12.85 22953706

[B69] McGwire B. S. Satoskar A. R. (2014). Leishmaniasis: clinical syndromes and treatment. *Int. J. Med.* 107 7–14. 10.1093/qjmed/hct116 PMC3869292 23744570

[B70] Mor A. (2009). Multifunctional host defense peptides: antiparasitic activities. *FEBS J.* 276 6474–6482. 10.1111/j.1742-4658.2009.07358.x 19817857

[B71] Moreira C. K. Rodrigues F. G. Ghosh A. Varotti F. P. Miranda A. Daffre S. (2007). Effect of the antimicrobial peptide gomesin against different life stages of *Plasmodium* spp. *Exp. Parasitol.* 116 346–353. 10.1016/j.exppara.2007.01.022 17376436 PMC1978196

[B72] Nakamura T. Furunaka H. Miyata T. Tokunaga F. Muta T. Iwanaga S. (1988). Tachyplesin, a class of antimicrobial peptide from the hemocytes of the horseshoe-crab (*Tachypleus tridentatus*) - isolation and chemical-structure. *J. Biol. Chem.* 263 16709–16713. 3141410

[B73] Ngwa C. J. Scheuermayer M. Mair G. R. Kern S. Brügl T. Wirth C. C. (2013). Changes in the transcriptome of the malaria parasite *Plasmodium falciparum* during the initial phase of transmission from the human to the mosquito. *BMC Genomics* 14:256 10.1186/1471-2164-14-256 PMC3640944 23586929

[B74] Olliaro P. Vaillant M. T. Sundar S. Balaseagaram M. (2012). More efficient ways of assessing treatments for neglected tropical diseases are required: innovative study designs, new endpoints, and markers of effects. *PLoS Negl. Trop. Dis.* 6:e1545 10.1371/journal.pntd.0001545 PMC3362612 22666508

[B75] Paes N. A. Silva L. A. (1999). Doenças infecciosas e parasitárias no Brasil: uma década de transição. *Rev Panam Salud Publica/Pan. Am. J. Public Health* 6 99–109. 10.1590/S1020-49891999000700004 10574011

[B76] Papadopoulou C. Kostoula A. Dimitriou D. Panagiou A. Bobojianni C. Antoniades G. (2005). Human and canine leishmaniasis in asymptomatic and symptomatic population in Northwestern Greece. *J. Infect.* 50 53–60. 10.1016/j.jinf.2004.05.004 15603841

[B77] Parada H. Carrasco H. A. Anez N. Fuenmayor C. Inglessis I. (1997). Cardiac involvement is a constant finding in acute Chagas disease: a clinical, parasitological and histopathological study. *Int. J. Cardiol.* 60 49–54. 10.1016/S0167-5273(97)02952-5 9209939

[B78] Pelegrini P. B. Quirino B. F. Franco O. L. (2007). Plant cyclotides: na unusual class of defense compounds. *Peptides* 28 1475–1481. 10.1016/j.peptides.2007.04.025 17586088

[B79] Powers J. P. S. Hancock R. E. W. (2003). The relationship between peptide structure and antibacterial activity. *Peptides* 24 1681–1691. 10.1016/j.peptides.2003.08.023 15019199

[B80] Public Health England (2014). *Malaria: Guidance, Data and Analysis.* Available at https://www.gov.uk/government/collections/malaria-guidance-data-and-analysis [accessed October 19, 2014].

[B81] Raghuraman H. Chattopadhyay A. (2007). Melittin: a membrane-active peptide with diverse functions. *Biosci. Rep.* 27 189–223. 10.1007/s10540-006-9030-z 17139559

[B82] Ramasamy R. (2014). Zoonotic malaria – global overview and research and policy needs. *Front. Public Health* 2:123 10.3389/fpubh.2014.00123 PMC4135302 25184118

[B83] Rangel M. Cabrera M. P. S. Kazuma K. Ando K. Wang X. Kato M. (2011). Chemical, and biological characterization of four new linear cationic α-helical peptides from the venoms of two solitary eumenine wasps. *Toxicon* 57 1081–1092. 10.1016/j.toxicon.2011.04.014 21549739

[B84] Rosendaal J. A. (1997). *Vector Control: Methods for Use by Individuals, and Communities* Chap. 3 Geneva: World Health Organization.

[B85] Segunpta D. Leonatiadou H. Mark A. E. Marrink S.-J. (2008). Toroidal pores formed by antimicrobial peptides show significant disorder. *Biochim. Biophys. Acta* 1178 2308–2317. 10.1016/j.bbamem.2008.06.007 18602889

[B86] Sforça M. L. Oyama S. Jr. Canduri F. Lorenzi C. C. B. Pertinez T. A. Konno K. (2004). How C-terminal carboxyamidation alters the mast cell degranulating activity of peptides from the venom of the eumenine solitary wasp. *Biochemistry* 43 5608–5617. 10.1021/bi0360915 15134435

[B87] Shen D. Wu B. (2014). Structure, biological properties and utilities of marine-derived antimicrobial peptides. *Curr. Organ. Chem.* 18 793–803.

[B88] Souza W. (2010). *Doenças Negligenciadas.* Rio de Janeiro: Academia Brasileira de Ciências 56.

[B89] Stark W. J. (2011). Nanoparticles in biological systems. *Angewandre Chem. Int. Edn.* 50 1242–1258. 10.1002/anie.200906684 21290491

[B90] Sundar S. Sinha P. K. Rai M. Verma D. K. Nawin K. Alam S. (2011). Comparison of short-course multidrug treatment with standard therapy for visceral leishmaniasis in India: an open label, non-inferiority, randomized controlled trial. *Lancet* 377 477–486. 10.1016/S0140-6736(10)62050-8 21255828

[B91] Tam J. P. Wang S. Wong K. H. Tan W. L. (2015). Antimicrobial peptides from plants. *Pharmaceuticals (Basel)* 8 711–757. 10.3390/ph8040711 26580629 PMC4695807

[B92] Tanaka H. Ishibashi J. Fujita K. Nakajima Y. Sagisaka A. Tomimoto K. (2008). A genome-wide analysis of genes and gene families involved in innate immunity of *Bombyx mori*. *Insect Biochem. Mol. Biol.* 38 1087–1110. 10.1016/j.ibmb.2008.09.001 18835443

[B93] Tarun A. S. Peng X. Dumpit R. F. Ogata Y. Rivera-Silva H. Camargo N. (2008). A combined transcriptome and proteome survey of malaria parasite liver stages. *Proc. Natl. Acad. Sci. U.S.A.* 105 305–310. 10.1073/pnas.0710780104 18172196 PMC2224207

[B94] Teixeira C. Vale N. Pérez B. Gomes A. Gomes J. R. B. Gomes P. (2014). “Recycling” classical drugs for malária. *Chem. Rev.* 114 11164–11220. 10.1021/cr500123g 25329927

[B95] Tian C. Gao B. Rodriguez Mdel C. Lanz-Mendoza H. Ma B. Zhu S. (2008). Gene expression, antiparasitic activity, and functional evolution of the drosomycin family. *Mol. Immunol.* 45 3909–3916. 10.1016/j.molimm.2008.06.025 18657321

[B96] Torrent M. Pulido D. Rivas L. Andreu D. (2012). Antimicrobial peptide action on parasites. *Curr. Drug Targets* 13 1138–1147. 10.2174/138945012802002393 22664071

[B97] Uniting to Combat NTDs (2014). *NTD Report 2014: Delivering on Promises and Driving Progress.* London: Uniting to Combat NTDs 46 Available at: http://unitingtocombatntds.org/sites/default/files/document/NTD%20Report%20Final%20%281%29.pdf

[B98] Vale N. Aguiar L. Gomes P. (2014). Antimicrobial peptides: a new class of antimalarial drugs. *Front. Phamacol.* 5:275 10.3389/fphar.2014.00275 PMC4271771 25566072

[B99] Verma N. Karmakar M. Singh K. P. Smita S. (2013). Structural and dynamic insights into S100B protein activity inhibition by melittin for the treatment of epilepsy. *Int. J. Comp. App. NSAAILS* 1 55–60.

[B100] Visser B. J. van Vugt M. Grobusch M. P. (2014). Malaria: an update on current chemotherapy. *Expert Opin. Pharmacother.* 15 2219–2254. 10.1517/14656566.2014.944499 25110058

[B101] Vizioli J. Bulet P. Hoffmann J. A. Kafatos F. C. Muller H. M. Dimopoulos G. (2001). Gambicin: a novel immune responsive antimicrobial peptide from the malaria vector *Anopheles gambiae*. *Proc. Natl. Acad. Sci. U.S.A.* 98 12630–12635. 10.1073/pnas.221466798 11606751 PMC60105

[B102] White N. J. (2004). Antimalarial drug resistance. *J. Clin. Invest.* 113 1084–1092. 10.1172/JCI21682 15085184 PMC385418

[B103] Whitebread S. Hamon J. Bojanic D. Urban L. (2005). Keynote review: in vitro safety pharmacology profiling: an essential tool for successful drug development. *Drug Discov. Today* 10 1421–1433. 16243262 10.1016/S1359-6446(05)03632-9

[B104] Whyllie S. Forth B. J. Kelner A. Sokolova A. Y. Berriman M. Farlamb A. H. (2015). Nitroheterocyclic drug resistance mechanisms in *Ttrypanosoma brucei*. *J. Antimicrob. Chemother.* [Epub ahead of print]. 10.1093/jac/dkv376 PMC4743696 26581221

[B105] World Health Organization [WHO] (2014). *Chagas Disease (American Trypanosomiasis): Fact Sheet n. 340.* Available at: http://www.who.int/mediacentre/factsheets/fs340/en/ [accessed October 20, 2014].

[B106] World Health Organization [WHO] (2015a). *Investing to Overcome the Global Impact of Neglected Tropical Diseases: The Who Report on Neglected Tropical Diseases.* Geneva: World Health Organization 211.

[B107] World Health Organization [WHO] (2015b). *Leishmaniasis. Media Center. Fact Sheet n. 375.* Available at: http://www.who.int/mediacentre/factsheets/fs375/en/ [accessed December 17th, 2015].

[B108] World Malaria Report (2015). *WHO Library Cataloguing-in-Publication Data.* WHO: Geneva 280.

[B109] Yang D. Chertov O. Oppenheim J. J. (2001). Participation of mammalian defensins and cathelicidins in anti-microbial immunity: receptors and activities of human defensins and cathelicidin (LL-37). *J. Leukoc. Biol.* 69 691–697. 11358975

[B110] Yang S. Carrasquer G. (1997). Effect of melittin on ion transport across cell membranes. *Zhongguo Yao Li Xue Bao* 18 3–5. 10072885

